# Extracellular Hsp90α Promotes Tumor Lymphangiogenesis and Lymph Node Metastasis in Breast Cancer

**DOI:** 10.3390/ijms22147747

**Published:** 2021-07-20

**Authors:** Qiaoyun Hou, Shuohua Chen, Qi An, Boya Li, Yan Fu, Yongzhang Luo

**Affiliations:** 1 Cancer Biology Laboratory, School of Life Sciences, Tsinghua University, Beijing 100084, China; hqy16@mails.tsinghua.edu.cn (Q.H.); chen-sh16@mails.tsinghua.edu.cn (S.C.); aq18@mails.tsinghua.edu.cn (Q.A.); liby15@mails.tsinghua.edu.cn (B.L.); 2 Beijing Key Laboratory for Protein Therapeutics, Tsinghua University, Beijing 100084, China; 3 The National Engineering Laboratory for Anti-Tumor Protein Therapeutics (NEL), Tsinghua University, Beijing 100084, China

**Keywords:** plasma Hsp90α, tumor biomarker, breast cancer, lymph node metastasis, extracellular Hsp90α, lymphangiogenesis

## Abstract

Early detection and discovery of new therapeutic targets are urgently needed to improve the breast cancer treatment outcome. Here we conducted an official clinical trial with cross-validation to corroborate human plasma Hsp90α as a novel breast cancer biomarker. Importantly, similar results were noticed in detecting early-stage breast cancer patients. Additionally, levels of plasma Hsp90α in breast cancer patients were gradually elevated as their clinical stages of regional lymph nodes advanced. In orthotopic breast cancer mouse models, administrating with recombinant Hsp90α protein increased both the primary tumor lymphatic vessel density and sentinel lymph node metastasis by 2 and 10 times, respectively. What is more, Hsp90α neutralizing antibody treatment approximately reduced 70% of lymphatic vessel density and 90% of sentinel lymph node metastasis. In the in vitro study, we demonstrated the role of extracellular Hsp90α (eHsp90α) as a pro-lymphangiogenic factor, which significantly enhanced migration and tube formation abilities of lymphatic endothelial cells (LECs). Mechanistically, eHsp90α signaled to the AKT pathway through low-density lipoprotein receptor-related protein 1 (LRP1) to upregulate the expression and secretion of CXCL8 in the lymphangiogenic process. Collectively, this study proves that plasma Hsp90α serves as an auxiliary diagnosis biomarker and eHsp90α as a molecular mediator promoting lymphangiogenesis in breast cancer.

## 1. Introduction

Breast cancer is the most frequently diagnosed malignant disease and the leading cause of cancer mortality in women worldwide [1]. For these patients, tumor metastasis at distant sites is the main cause of cancer-related death instead of the primary tumor [2]. Evidenced by considerable clinicopathological observations, most carcinomas, especially breast cancer, preferentially utilize lymph vessels as the initial conduit for tumor dissemination [3,4]. Such spread of disseminated tumor cells converges to regional lymph nodes, which serve as potential reservoirs for further distant metastases via the circulatory system [5]. With currently available therapy approaches, the overall survival of patients with metastatic breast cancer is less than 5%. However, the early-stage breast cancer is potentially curable [6]. Therefore, early detection and discovery of new therapeutic targets are urgently needed to lower the rates of breast cancer metastasis and mortality.

Hsp90α is an essential intracellular molecular chaperone for hundreds of protein clients. The diversity and importance of its clients make Hsp90α a central regulator of cellular processes ranging from protein folding to DNA repair, development, immune response and many other important processes [7]. Recently, Hsp90α has been demonstrated to localize extracellularly, where it facilitates tumor invasion and metastasis [8,9,10]. In line with these findings, our team has found levels of secreted Hsp90α are significantly elevated in cancer patients’ plasma, and are positively correlated with tumor malignancy [11]. Considerable efforts have been made to translate this discovery into the clinic. Two large-scale clinical trials have proven that plasma Hsp90α is an excellent diagnostic biomarker for lung cancer [12,13] and liver cancer [14]. The plasma concentrations of Hsp90α were noted to be higher in breast cancer patients than the control group [11,15], however there are currently no clinical trials to investigate whether plasma Hsp90α could be applied to the auxiliary diagnosis of breast cancer.

Mounting preclinical studies and clinical research has revealed that lymphangiogenesis, the growth of lymphatic vessels from preexisting LECs in primary tumors, increases the delivery channels for tumor cell dissemination [16,17]. Correspondingly, the density of lymphatic vessels correlates with the occurrence of lymph node metastasis and adverse clinical outcome in many human cancers [18]. Therefore, targeting primary lymphangiogenesis is an important strategy for the treatment of metastatic breast cancer. Vascular endothelial growth factor C (VEGF-C) and VEGF-D are the best characterized lymphangiogenic factors, which function via their receptor VEGFR-3 on the membrane of LECs [19,20,21]. In addition, a series of other lymphangiogenic factors and corresponding receptors have also been reported [22]. For instance, CXCL12/CXCR4 pathway promotes the migration and tube formation of LECs in vitro, and directly induces lymphangiogenesis in vivo [23]. When it comes to eHsp90α, our team has confirmed that eHsp90α induces angiogenesis during wound healing and cancer progression [24,25]. To date, however, no research has been reported the biological function and molecular mechanism of eHsp90α in the coordinated processes of lymphangiogenesis.

Here we conducted an official, large-scale, and multicenter clinical trial to qualify plasma Hsp90α as a novel diagnostic biomarker for breast cancer, which has considerable value in the early detection of breast cancer. Moreover, levels of plasma Hsp90α in breast cancer patients gradually increased as the spread of tumor cells to regional lymph nodes intensified, implying its participation in lymphatic metastasis. In the experimental study, we observed that eHsp90α induced lymphangiogenesis and lymph node metastasis in breast cancer, and further investigated the molecular mechanism of eHsp90α being a pro-lymphangiogenic factor. Our work on eHsp90α provides not only an auxiliary diagnostic biomarker for breast cancer, but also a potential target for therapeutic intervention in pathogenic lymphangiogenesis.

## 2. Results

### 2.1. Plasma Hsp90α Is a Novel Diagnosis Biomarker for Breast Cancer

**Figure 1:**
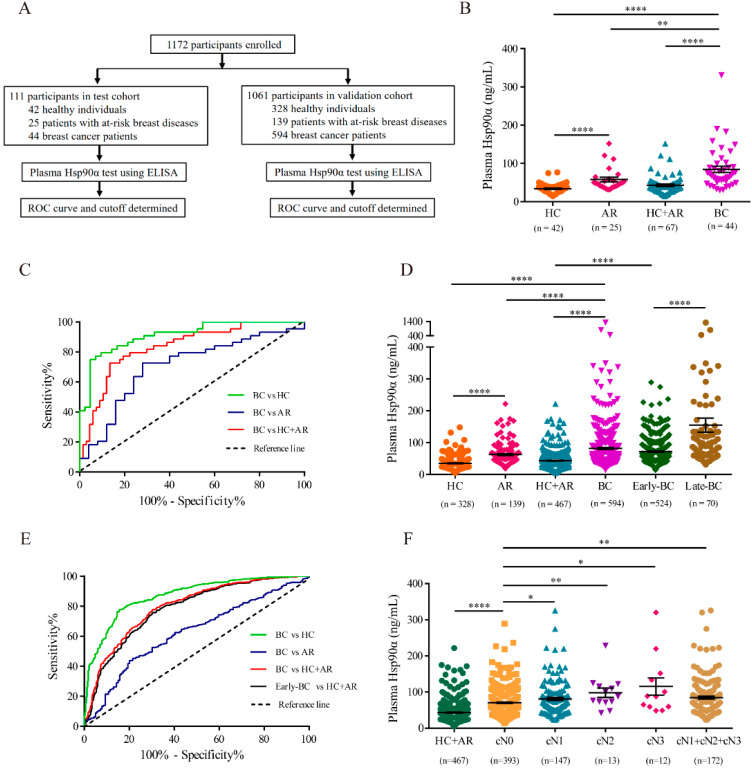
Plasma Hsp90α is a novel diagnosis biomarker for breast cancer. (**A**) Enrollment of participants in test cohort and validation cohort. (**B**) Plasma concentrations of Hsp90α in breast cancer patients and different controls in test cohort. (**C**) ROC curve of plasma Hsp90α for breast cancer patients versus different controls in test cohort. (**D**) Plasma Hsp90α levels in breast cancer patients and different control groups in validation cohort. (**E**) ROC curve of plasma Hsp90α for patients with breast cancer versus different control groups in validation cohort. (**F**) Plasma levels of Hsp90α of breast cancer patients in different clinical stages of regional lymph node metastasis. BC indicates breast carcinoma. HC is short for healthy control. AR represents at-risk, non-cancerous breast diseases. Non-BC indicates non-cancer control, including HC and AR groups. Early-BC and Late-BC stand for patients with early-stage (stages I and II) breast cancer and late-stage (stages III and IV) breast cancer, respectively. AUC represents area under curve. CI is short for confidence interval. Data are shown as mean ± SEM. * *p* < 0.05; ** *p* < 0.01; **** *p* < 0.0001.

To explore the performance of plasma Hsp90α in breast cancer auxiliary diagnosis, 111 participants, including 44 breast cancer patients, 25 patients with at-risk breast diseases and 42 healthy individuals, were enrolled from hospitals into the test cohort (Figure 1A and Supplementary Table S1). Blood samples were collected and concentrations of plasma Hsp90α were measured by ELISA. Plasma Hsp90α values were markedly elevated in breast cancer patients (median 68.83 ng/mL, mean 84.41 ng/mL) compared to those in the non-cancer control (healthy individuals plus patients with at-risk breast diseases; median 38.09 ng/mL, mean 42.89 ng/mL) and those in the at-risk group (Figure 1B and Supplementary Table S4). The ROC curve determined 49.11 ng/mL as the best cutoff value to detect breast cancer from the non-cancer control (AUC 0.8351, sensitivity 79.55%, specificity 77.61%; Figure 1C and Table 1).

To confirm these results, we continued to recruit 1061 participants (594 breast cancer patients, 139 patients with at-risk breast diseases and 328 healthy individuals) into the validation cohort (Figure 1A and Supplementary Table S1). Likewise, plasma Hsp90α concentrations were at significantly higher levels in patients with breast cancer (median 66.50 ng/mL, mean 81.87 ng/mL) than those in the non-cancer group (median 38.44 ng/mL, mean 43.32 ng/mL), and the values were positively correlated with cancer malignancy (Figure 1D and Supplementary Table S5). When using the optimum diagnostic cutoff value 50.29 ng/mL, plasma Hsp90α could distinguish breast cancer patients with a sensitivity of 74.41% and a specificity of 71.95% from the non-cancer control (AUC 0.7973; Figure 1E and Table 1). Considering the importance of early detection to breast cancer treatment, we next evaluated the diagnostic performance of plasma Hsp90α in discriminating early-stage breast cancer. An obvious increase of plasma Hsp90α concentrations in patients with early-stage breast cancer (stages I and II; median 64.53 ng/mL, mean 72.11 ng/mL) was noticed compared with those in the non-cancer group (Figure 1D and Supplementary Table S5). Furthermore, ROC curve identified plasma Hsp90α (AUC 0.7872, sensitivity 72.33%, specificity 71.95%, optimal cutoff 50.29 ng/mL) as a promising biomarker for differentiating early-stage breast cancer patients from the non-cancer control (Figure 1E and Table 1).

In the case of breast cancer, lymph node metastasis significantly contributes to breast cancer progression, and the presence of metastatic tumor cells in regional lymph nodes is regarded as an indicator of poor prognosis [26]. To investigate the role of Hsp90α in lymph node metastasis, 565 breast cancer patients with specific information regarding clinical stages of regional lymph nodes metastasis (cN) from the validation cohort were chosen. cN stages (cN0, cN1, cN2, cN3) were classified by clinicians according to American Joint Committee on Cancer (AJCC) TNM staging for breast cancer [27]. It was noticed that the plasma Hsp90α concentrations gradually increased as the spread of tumor cells to regional lymph nodes intensified (from cN0 to cN3, Figure 1F and Supplementary Table S6). In general, concentrations of plasma Hsp90α were significantly higher in patients with regional lymph node metastasis (cN1 + cN2 + cN3, median 71.63 ng/mL, mean 84.64 ng/mL) than those without lymph node metastasis (cN0, median 64.13 ng/mL, mean 70.74 ng/mL). Altogether, our clinical data not only qualify plasma Hsp90α as a novel diagnostic biomarker for breast cancer, but also indicate the involvement of eHsp90α in the lymphatic metastasis process of breast cancer.

### 2.2. eHsp90α Facilitates Lymph Node Metastasis and Primary Tumor Lymphangiogenesis in Breast Cancer In Vivo

**Figure 2:**
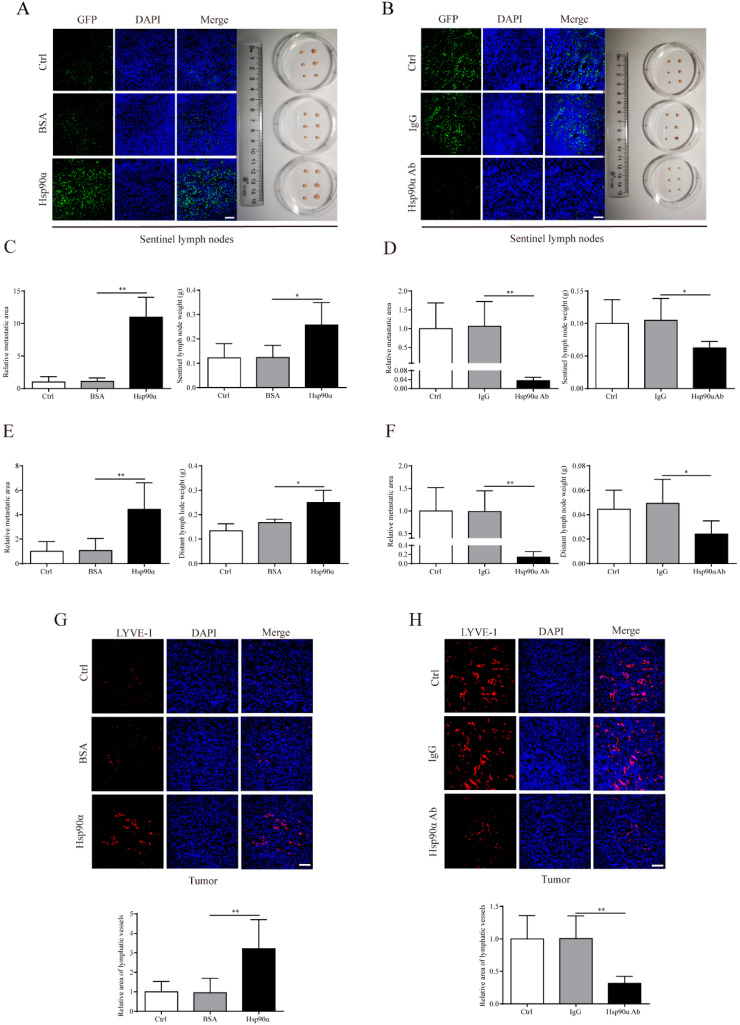
eHsp90α facilitates lymph node metastasis and primary tumor lymphangiogenesis in breast cancer in vivo. (**A**) Representative IF images of metastatic tumor cells (GFP labeled) in sentinel lymph nodes removed from MCF-7/GFP tumor-bearing mice in different groups were displayed (left). Scale bar, 50 μm. Dissected sentinel lymph nodes were photographed (right). (**B**) Representative IF images of metastatic tumor cells (GFP labeled) in sentinel lymph nodes removed from MDA-MB-231/GFP tumor-bearing mice in different groups were displayed (left). Scale bar, 50 μm. Dissected sentinel lymph nodes were photographed (right). (**C**,**D**) Quantification results of relative sentinel lymph node metastatic area (left) and sentinel lymph node weight (right) from MCF-7/GFP orthotopic mouse models (**C**) and MDA-MB-231/GFP orthotopic mouse models (**D**). (**E**,**F**) Quantification results of relative distant lymph node metastatic area (left) and distant lymph node weight (right) from MCF-7/GFP orthotopic mouse models (**E**) and MDA-MB-231/GFP orthotopic mouse models (**F**). (**G**) Representative IF images of lymphatic vessels (red staining of LYVE-1) in MCF-7/GFP primary tumors from different groups were displayed (top). Scale bar, 100 μm. Quantification results of relative lymphatic vessels density were shown (bottom). (**H**) Representative IF images of lymphatic vessels (red staining of LYVE-1) in MDA-MB-231/GFP primary tumors from different groups were displayed (top). Scale bar, 100 μm. Quantification results of relative lymphatic vessels density were shown (bottom). Data are displayed as mean ± SD. n = 6. * *p* < 0.05; ** *p* < 0.01.

To further investigate the functional relevance of eHsp90α in the process of lymph node metastasis, two independent orthotopic breast cancer mouse models were constructed, of which MCF-7/GFP cells or MDA-MB-231/GFP cells were implanted into the mammary fat pads. Considering MCF-7 is an Hsp90α low-secretion cell line and MDA-MB-231 secretes relatively high level of Hsp90α [11], we treated mice bearing MCF-7/GFP with recombinant Hsp90α protein and treated MDA-MB-231/GFP implanted mice with Hsp90α neutralizing antibody, respectively. It was found that recombinant Hsp90α protein or Hsp90α neutralizing antibody had no effect on tumor growth and tumor weight (Supplementary Figure S1A,B), which was consistent with our previous finding [11]. However, recombinant Hsp90α protein administration increased the sentinel lymph node metastasis area by 10 times compared with the PBS or BSA administration in the MCF-7/GFP mouse models (Figure 2A,C). More strikingly, in the MDA-MB-231/GFP mouse models, Hsp90α neutralizing antibody treatment reduced more than 90% area of sentinel lymph node metastasis by comparison with the PBS or IgG treatment (Figure 2B,D). In the aspect of distant lymph node metastasis, similar results were observed (Figure 2E,F; Supplementary Figure S1C,D).

It is well known that primary tumor lymphangiogenesis facilitates tumor dissemination to lymph nodes [28], thus we speculated that eHsp90α might function through inducing primary tumor lymphangiogenesis to promote lymph node metastasis. LYVE-1 (lymphatic vessel hyaluronan receptor-1), one of the most widely used and specific markers for lymphatic endothelial cells [29], was fluorescently labeled to measure the primary tumor lymphatic vessel density. Quantification of LYVE-1 positive areas in MCF-7/GFP primary tumor tissues demonstrated that the primary tumor lymphatic vessel density in the recombinant Hsp90α protein group increased by two times compared with that in the control group (Figure 2G). A dramatic decrease of lymphatic vessel density (nearly 70%) was also observed in MDA-MB-231/GFP tumors when treated with Hsp90α neutralizing antibody (Figure 2H). Collectively, these immunofluorescent staining analysis results reveal that eHsp90α promotes primary tumor lymphangiogenesis and lymph node metastasis in the orthotopic breast cancer mouse models.

### 2.3. eHsp90α Enhances Migration and Tube Formation Abilities of LECs

**Figure 3:**
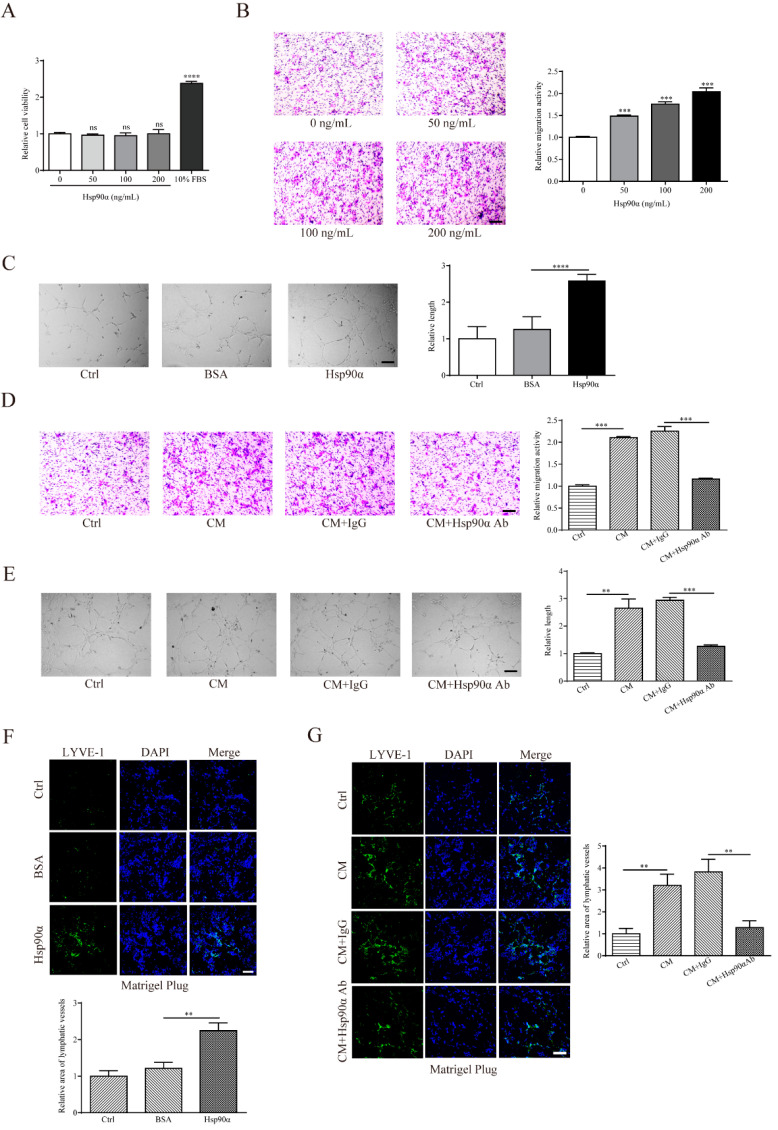
eHsp90α enhances migration and tube formation abilities of LECs. (**A**) Quantification results of the cell viability of hLECs treated with recombinant Hsp90α protein at different dosages in the culture medium containing 1% FBS. Culture medium with 10% FBS was used as the positive control. (**B**,**C**) Representative images and quantification results of the cell migration ability (**B**) and the tube formation ability (**C**) of hLECs treated with indicated reagents (200 ng/mL Hsp90α or control BSA for the tube formation assay). Scale bar, 200 μm. (**D**,**E**) Representative images and quantification results of the cell migration ability (**D**) and the tube formation ability (**E**) of hLECs in the presence of MDA-MB-231 CM, which was pre-mixed with control IgG or Hsp90α neutralizing antibody (200 ng/mL) for 30 min at 37 °C. Scale bar, 200 μm. (**F**,**G**) Matrigel plugs containing PBS, BSA or recombinant Hsp90α protein (**F**) and plugs containing MDA-MB-231 CM, which was pre-incubated with control IgG or Hsp90α neutralizing antibody (**G**) were subcutaneously injected into BALB/c mice. After 8 days, plugs were applied to IF analysis. Representative images of lymphatic vessel (green staining of LYVE-1) were displayed (top). Quantification results were shown (bottom). Concentration of recombinant Hsp90α protein or Hsp90α neutralizing antibody is 1 μg/mL. Scale bar, 100 μm. Data from three independent experiments are represented as mean ± SD. ** *p* < 0.01; *** *p* < 0.001; **** *p* < 0.0001.

To explore the regulatory mechanism of eHsp90α in lymphangiogenesis, we assessed LEC proliferation, migration, and tube formation, all of which are essential cellular events in the coordinated lymphangiogenic processes [30]. It was shown that exogenous recombinant Hsp90α protein had no detectable influence on the human LECs proliferation (Figure 3A). However, it significantly promoted migration and tube formation of hLECs (Figure 3B,C). To further evaluate the effect of tumor-associated eHsp90α in lymphangiogenesis, conditioned medium (CM) from MDA-MB-231 was collected. As we expected, MDA-MB-231 CM markedly augmented migration and tube formation activities of hLECs, while Hsp90α neutralizing antibody impeded the lymphangiogenic abilities stimulated by the CM (Figure 3D,E). Similar phenomena were noted in the mouse LECs (Supplementary Figure S2).

To confirm above in vitro observations, Matrigel plugs were subcutaneously planted into mice considering the accessibility of subcutis to abundant lymphatic vessels. We discovered that recombinant Hsp90α protein increased the lymphatic vessel area, indicated by the immunofluorescent staining area of LYVE-1 in the sections of planted plugs (Figure 3F). Similarly, MDA-MB-231 CM strengthened the lymphatic vessel formation ability in the Matrigel plugs, which could be weakened by the administration of Hsp90α neutralizing antibody (Figure 3G). Taken together, our data demonstrate that eHsp90α enhances the lymphangiogenic abilities both in vitro and in vivo.

### 2.4. eHsp90α Mediates Lymphangiogenic Activities through the LRP1-AKT Pathway

**Figure 4:**
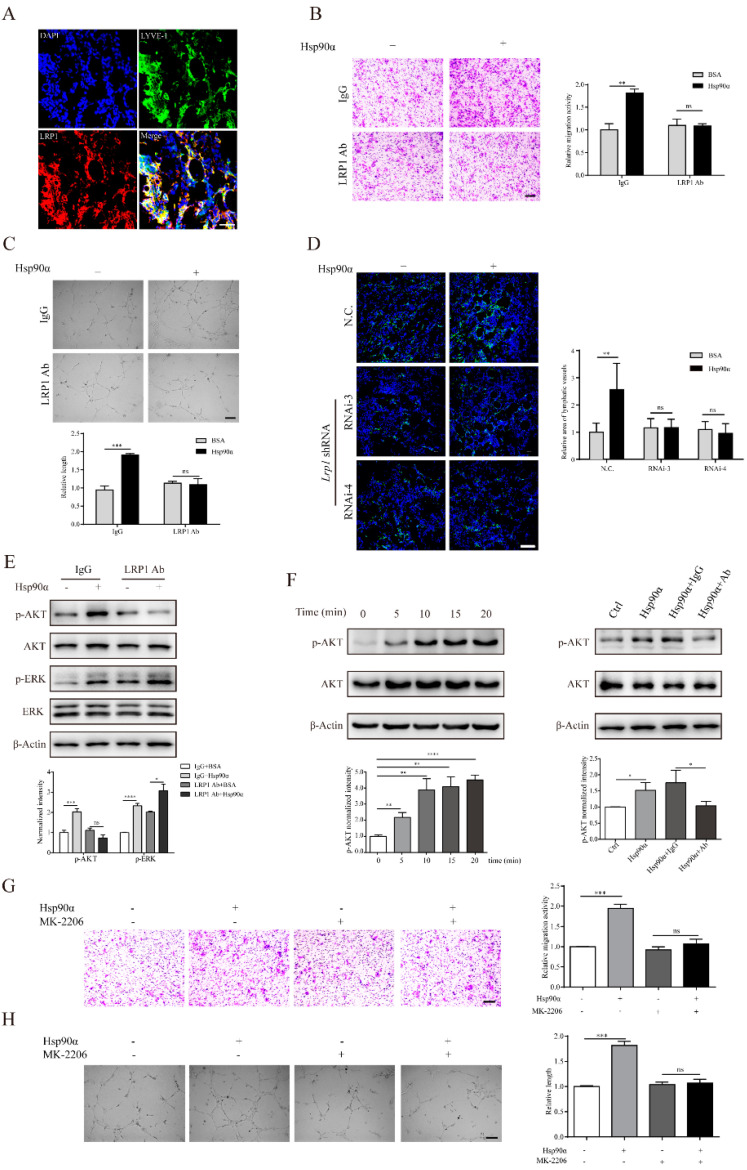
eHsp90α mediates lymphangiogenic activities through the LRP1-AKT pathway. (**A**) Detection of LRP1 (red staining) in the newly formed lymphatic vessels (indicated by LYVE-1, green staining) from the Matrigel plug assay by IF. Scale bar, 50 μm. (**B**,**C**) Representative images and quantification results of the migration ability (**B**) and tube formation ability (**C**) of hLECs induced by Hsp90α (200 ng/mL) when cells were pretreated with LRP1 neutralizing antibody (200 ng/mL). Scale bar, 200 μm. (**D**) Matrigel plugs containing BSA or Hsp90α protein (1 μg/mL) in the presence of lentivirus-delivered Lrp1 shRNA or negative control (2.5 × 10^6^ U) were subcutaneously injected into BALB/c mice. After 8 days, plugs were applied to IF analysis. Lymphatic vessel (green staining of LYVE-1) was displayed (left). Quantification results were shown (right). Scale bar, 100 μm. (**E**) hLECs were stimulated by recombinant Hsp90α protein in the presence of LRP1 neutralizing antibody or its isotype IgG control. The protein levels of p-AKT, p-ERK, AKT and ERK were measured by Western blotting. Quantitative results are shown below. (**F**) The protein levels of p-AKT and AKT in hLECs exposed to recombinant Hsp90α protein at indicated points of time were determined by Western blotting (left). The protein levels of p-AKT and AKT in hLECs treated with indicated regeants were detected by Western blot analysis (right). Quantitative results are shown below. (**G, H**) hLECs were pretreated with MK-2206 (inhibitor of AKT, 1 μM) or DMSO for 30 min. Representative images and quantification results of the cell migration ability (**G**) and tube formation ability (**H**) of inhibitor-pretreated hLECs induced by recombinant Hsp90α protein. Scale bar, 200 μm. Data are represented as mean ± SD. * *p* < 0.05; ** *p* < 0.01; *** *p* < 0.001; **** *p* < 0.0001.

It has been well reported that cell surface receptor LRP1 transmits eHsp90α signal intracellularly to stimulate cell motility during wound healing [31,32] and cancer invasion [33,34]. What is more, recent research has demonstrated that LRP1 is essential for vascular development and angiogenesis [35,36,37]. However, the existence and function of LRP1 in lymphatic endothelial cells remain to be determined. First of all, in the newly formed lymphatic vessels in the Matrigel plug assay, the prominent overlapping of LRP1 and LYVE-1 ascertained the presence of LRP1 in lymphatic vessels in vivo (Figure 4A). We also confirmed that LRP1 existed in the cultured hLECs and mLECs in vitro (Supplementary Figure S3A,B). What is more, the co-IP assay confirmed that eHsp90α could bind with LRP1 in hLECs (Supplementary Figure S3C). To find out whether the lymphangiogenic signal from eHsp90α was transmitted intracellularly by LRP1, we pretreated LECs with LRP1 neutralizing antibody. It was shown that eHsp90α could not enhance migration and tube formation activities of hLECs and mLECs when LRP1 was pre-blocked (Figure 4B,C; Supplementary Figure S3D,E). To further validate the necessity of LRP1 in the eHsp90α-mediated lymphangiogenic process in vivo, we added lentivirus-delivered Lrp1 shRNA into the Matrigel plugs. The RNA interference efficacy was pre-determined by Lrp1 siRNA transfection in mLECs in vitro (Supplementary Figure S3F). The immunofluorescence results illustrated that recombinant Hsp90α was unable to induce lymphangiogenesis in the Matrigel plugs in the presence of Lrp1 shRNA (Figure 4D). In sum, these results support that eHsp90α mediates lymphangiogenic activities through LRP1 in vitro and in vivo.

To investigate whether eHsp90α utilizes the cross-membrane signal transduction to execute its pro-lymphangiogenic function, we need to testify that LRP1 connects eHsp90α to intracellular signaling networks. It has been reported that the eHsp90α-LRP1 interaction gives rise to a series of promotility signal cascades, such as AKT and ERK activation to promote cell motility during wound healing and cancer progression [38]. Alitalo group has documented that induction of AKT and ERK phosphorylation is necessary for the lymphangiogenic activities mediated by VEGF-C/D [39]. To explore the downstream signal pathway of eHsp90α, we treated hLECs with Hsp90α protein in the presence of LRP1 neutralizing antibody. The Western blotting results showed that exogenous Hsp90α administration caused an obvious increase in the phosphorylation of AKT and ERK in hLECs (Figure 4E). However, the eHsp90α-induced phospho-AKT, but not phospho-ERK, was reduced when LRP1 was pre-blocked (Figure 4E), suggesting that the intracellular AKT cascade was the downstream of eHsp90α signal which was bridged by LRP1. Next, we focused on the AKT pathway in the eHsp90α-modulated lymphangiogenesis. The time-gradient analysis displayed that eHsp90α stimulation led to a sustained activation of AKT (Figure 4F). Besides, the increased phospho-AKT level could be inhibited by the Hsp90α neutralizing antibody (Figure 4F). More importantly, activation of AKT was crucial for the lymphangiogenic ability of eHsp90α. It was shown that activities of migration and tube formation induced by eHsp90α were impaired when the AKT signal cascade was inhibited by MK-2206 (Figure 4G,H; Supplementary Figure S3G). Collectively, we come to the conclusion that eHsp90α-LRP1 modulates lymphangiogenesis via the AKT signal pathway.

As mentioned earlier, VEGF-C and VEGF-D are the most studied lymphangiogenic factors. With the discovery of new pro-lymphangiogenic factor, it is natural to inquire into the relationship between the existing and emerging ones. For example, we found there was an additive promoting effect on hLEC migration when Hsp90α and VEGFC were simultaneously administrated (Supplementary Figure S4A). Additionally, it was reported that the lymphangiogenic functions of several pro-lymphangiogenic factors, such as VEGF-A, FGF (fibroblast growth factor) and HGF (hepatocyte growth factor) were at least partially impaired when VEGFR-3 was blocked [40,41,42]. Therefore, we treated hLECs with Hsp90α protein in the presence or absence of VEGFR-3 specific inhibitor, SAR131675. It was displayed that the ability of eHsp90α to promote hLEC migration was unaffected by SAR13167 (Supplementary Figure S4B), indicating the eHsp90α-mediated lymphangiogenic migration is independent of the VEGFR-3 pathway.

### 2.5. CXCL8 Functions in the eHsp90α-Induced Lymphangiogenic Process in hLECs

**Figure 5:**
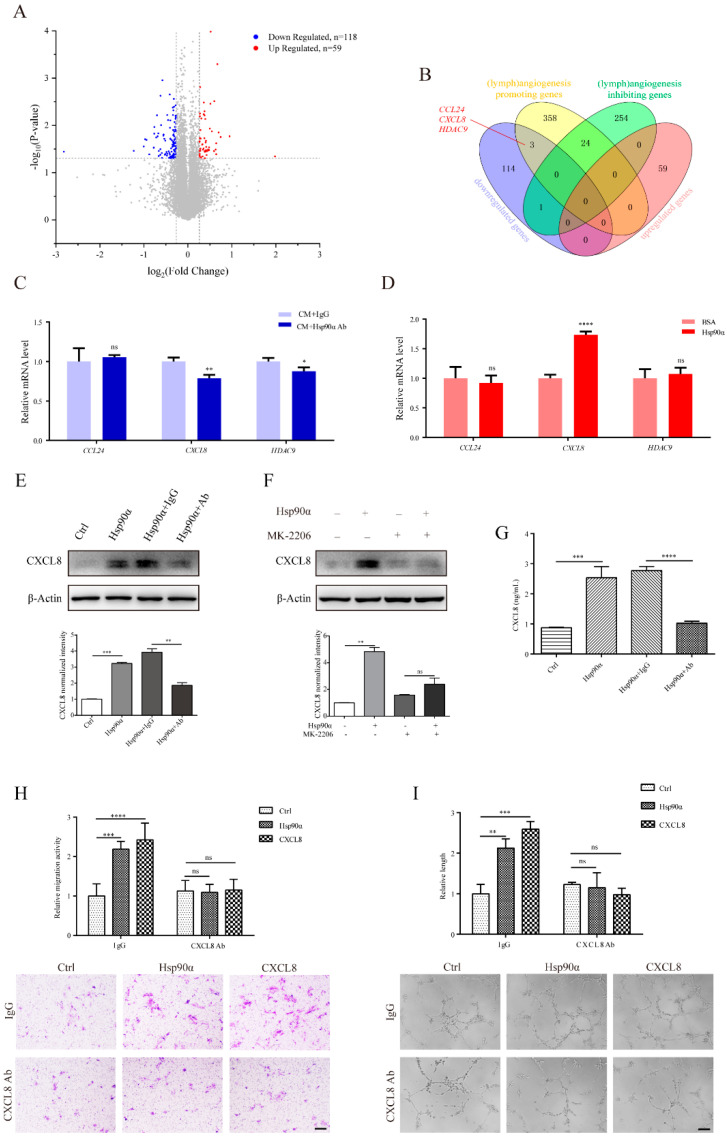
CXCL8 functions in the eHsp90α-induced lymphangiogenic process in hLECs. (**A**) The transcriptome levels of hLECs in the Hsp90α Ab group and the IgG control group were analyzed. Volcano plot was drawn to present the upregulated and downregulated DEGs by Hsp90α neutralizing antibody treatment. In total, 59 upregulated DEGs and 118 downregulated DEGs were colored in red and blue, respectively. (**B**) Venn diagram was drawn to show the overlapping genes between upregulated DEGs and negative-related lymphangiogenic genes, also between downregulated DEGs and positive-related lymphangiogenic genes. (**C**) Validation of RNA-seq results by qRT-PCR. (**D**) Further exploration of CCL24, CXCL8, HDAC9 expression in hLECs treated with recombinant Hsp90α protein by qRT-PCR. (**E**) Immunoblotting analyses of CXCL8 expression stimulated by recombinant Hsp90α protein in the presence of Hsp90α neutralizing antibody or control IgG. Quantitative results are shown below. (**F**) The protein level of CXCL8 induced by recombinant Hsp90α protein with or without MK-2206. Quantitative results are shown below. (**G**) Quantification of secreted CXCL8 levels in hLEC supernatant measured by ELISA. (**H**,**I**) Representative and quantified data showing the relative migration (**H**) and tube formation (**I**) activities of hLECs stimulated with recombinant Hsp90α protein (200 ng/mL) or CXCL8 protein (1 ng/mL) in the presence or absence of CXCL8 neutralizing antibody (10 ng/mL). Scale bar = 200 μm. Data are shown as mean ± SD. * *p* < 0.05; ** *p* < 0.01; *** *p* < 0.001; **** *p* < 0.0001.

To further investigate the downstream functional genes in the eHsp90α-induced lymphangiogenic process, we analyzed the transcriptome levels of hLECs under the influence of pre-processed MDA-MB-231 CM. In the Hsp90α Ab-treated group, CM was pre-neutralized with Hsp90α neutralizing antibody, and the same CM was pre-mixed with IgG in the IgG-treated group. Bioinformatics analyses concluded that 59 upregulated genes and 118 downregulated genes influenced by Hsp90α neutralizing antibody were chosen as differentially expressed genes (DEGs) (Figure 5A; Supplementary Table S7). In order to explore the functionality of these DEGs, Gene Ontology (GO) analysis was conducted. Chemokine activity and lymph vessel development, which were likely associated with lymphangiogenesis, were found among the top 20 enrichment pathways (Supplementary Figure S5A).

Subsequently, we made efforts to pinpoint the specific genes regulated by eHsp90α in the lymphangiogenic process of hLECs. Since the molecular control of lymphangiogenesis has partial similarities to that of angiogenesis [30], we compared DEGs with genes participating in the regulation of lymphangiogenesis and angiogenesis. What is more, considering the inhibitory effect of Hsp90α neutralizing antibody on lymphangiogenesis, we intersected downregulated DEGs with (lymph)angiogenesis promoting genes, also crossed upregulated DEGs with (lymph)angiogenesis inhibiting genes. The overlapping results revealed that three (lymph)angiogenesis promoting genes, CCL24 (C-C Motif Chemokine Ligand 24), CXCL8 (C-X-C Motif Chemokine Ligand 8) and HDAC9 (Histone Deacetylase 9) were downregulated in the Hsp90α Ab-treated group (Figure 5B). However, the qRT-PCR analysis exhibited that Hsp90α neutralizing antibody decreased the expression of CXCL8 and HDAC9 (Figure 5C). Besides, recombinant Hsp90α protein only increased the mRNA level of CXCL8, and the elevated CXCL8 expression could be inhibited by Hsp90α neutralizing antibody (Figure 5D,E; Supplementary Figure S5B). In addition, we observed a similar pattern on the expression of Cxcl1/2/6 (the surrogates of CXCL8 in murine [43]) in mLECs (Supplementary Figure S5C). Thus, in the following study, our focus was concentrated on CXCL8 in the eHsp90α-mediated lymphangiogenic process of hLECs.

CXCL8 is generally produced and secreted in response to several stimuli, including inflammatory cytokines, microbe products and cellular stresses. Initially recognized for its property of controlling leukocyte chemotaxis, secreted CXCL8 is now also known for its significant role in human cancer-related angiogenesis [44]. Apart from being the first described angiogenic chemokine [45], CXCL8 is proven to function as a strong promoter of lymphangiogenesis in recent research [46,47]. It has been reported that CXCL8 expression in lung epithelial cells is modulated by the PI3K/AKT pathway [48]. We also discovered that exogenous Hsp90α treatment could not upregulate the protein level of CXCL8 when the AKT inhibitor MK-2206 was administrated, establishing the eHsp90α-AKT-CXCL8 pathway in hLECs (Figure 5F). We next examined the secretion level of CXCL8 in order to explore the function of it in the eHsp90-induced lymphangiogenesis. As expected, the secretion of CXCL8 in hLEC supernatant was nearly augmented by two times by recombinant Hsp90α protein, which could be hampered by the administration of Hsp90α neutralizing antibody (Figure 5G). More importantly, we discovered that there was a remarkable reduction in the eHsp90α-enhanced migration and tube formation activities when CXCL8 was blocked, suggesting the necessity of CXCL8 in the lymphangiogenic process induced by eHsp90α (Figure 5H,I). In sum, CXCL8 is upregulated by eHsp90α via activation of the AKT signal pathway and functions in the eHsp90α-induced lymphangiogenic process.

## 3. Discussion

Breast cancer has now surpassed lung cancer being the primary cause of cancer incidence, and also the fifth leading origin of cancer death worldwide [49]. Early detection is crucial for alleviating the heavy burden of breast cancer. Here, we verified plasma Hsp90α as an auxiliary diagnostic biomarker for breast cancer, especially for early-stage breast cancer. Plasma Hsp90α can distinguish 72.33% of early-stage breast cancer patients from healthy people and patients with at-risk breast diseases. Apart from early detection, improving our understanding to the mechanism of lymphatic metastasis will provide new therapeutic targets for the metastatic breast cancer. It has been demonstrated that lymphangiogenesis is positively associated with lymphatic metastasis, and negatively correlated with survival in breast cancer patients [50]. In the present study, we reveal for the first time the role of eHsp90α as a novel pro-lymphangiogenic molecule, which increases lymphatic vessel densities in primary tumors in vivo and promotes lymphangiogenic activities of LECs in vitro.

In cancer cells, the necessary guardian function of Hsp90α as an intracellular molecular chaperone is subverted to stabilize activated oncoproteins and buffer cellular stress to allow malignant transformation [51]. Recently, it has been widely reported that Hsp90α can be constitutively secreted by a variety of tumor cell lines, including breast, colon, bladder, skin, prostate, ovary, liver and brain [52]. The secreted eHsp90α is shown to be crucial for the regulation of cancer invasiveness and metastasis [38]. We recently proved that eHsp90α contributes to the establishment of pre-metastatic environment in the mouse lung before the arrival of melanoma cells (to be submitted), supporting the feasibility of early detection of cancer using eHsp90α. Besides, our team has developed a quantitative ELISA kit for plasma Hsp90α, which was approved by the China Food and Drug Administration (CFDA) applied to the lung and liver cancers. Plasma Hsp90α exhibits a remarkable performance in the diagnosis of early-stage liver cancer [14]. In our present study, we extend the application of plasma Hsp90α to the diagnosis of breast cancer. The most widely used blood biomarker for breast cancer is CA15-3 (cancer antigen 15-3), whose clinical use is to monitor therapy in patients with metastatic disease. However, the main limitation of CA15-3 is that its serum levels are rarely elevated in early-stage breast cancer patients [53]. In the aspect of blood biomarker for the early detection of breast cancer, our work on plasma Hsp90α fills the void. Actually, we recently have randomly extracted partial data from several clinical trials and analyzed the plasma values of Hsp90α from patients with different types of cancer (including liver, lung, colorectal, breast, stomach, pancreatic, esophagus cancer or lymphoma). The results led us to the conclusion that plasma Hsp90α is qualified as a pan-cancer biomarker [15]. Inevitably, the clinical application of plasma Hsp90α will be a powerful measure to achieve early diagnosis of cancer and thus reduce the cancer mortality.

When breast cancer cells begin to spread, lymph nodes are often the first station. It has been clearly presented that cancer cells in lymph nodes directly invade lymph node blood vessels to enter the bloodstream before colonizing distant organs [54,55]. In our present clinical study, concentrations of plasma Hsp90α in breast cancer patients gradually increased as their clinical stages of regional lymph nodes advanced, indicating the involvement of eHsp90α in the lymph node metastasis. It is noteworthy that the concentration increase from non-cancer control to cN0 breast cancer is also obvious, suggesting eHsp90α initially functions before the advent of lymph node metastasis. The phenomenon that eHsp90α promotes lymph node metastasis might be explained by its reported mechanisms on extracellular matrix (ECM) degradation [8,11], tumor epithelial mesenchymal transition (EMT) activation [56,57,58] and so on. However, we paid attention to the direct influence of eHsp90α on the lymphatic system in this study. The new role of eHsp90α as a pro-lymphangiogenic factor to promote lymph node metastasis will enrich our knowledge about the tremendous influences of eHsp90α on tumor metastasis.

The main function of eHsp90α is to promote cell motility, which can be achieved by two non-exclusive manners. One is to activate its extracellular clients, and the other is to interact with its surface receptors. Matrix metalloproteinase-2 (MMP-2), one extracellular client of eHsp90α, participates in the eHsp90α-induced angiogenesis [25]. Lysyl Oxidase-like protein 2 (LOXL2), another extracellular client of eHsp90α, was recently proven to be a pro-lymphangiogenic factor [59]. It is reasonable to speculate that eHsp90α might modulate lymphangiogenesis via interacting with MMP-2 to break down ECM or with LOXL2 to stiffen ECM. However, the involvement of extracellular clients in the eHsp90α-mediated lymphangiogenesis remains to be determined. In this research, eHsp90α is regarded as an extracellular signal molecule to transmit its pro-lymphangiogenic signal intracellularly via its surface receptor LRP1. The eHsp90α-LRP1 interaction activates the AKT motility signal pathway in LECs to promote lymphangiogenic migration and tube formation. However, it is possible that AKT is not the direct target of LRP1 in the eHsp90α-modulated lymphangiogenic process (Supplementary Figure S3H). Intracellular adaptor proteins and kinases are likely to be required for the LRP1-dependent AKT phosphorylation [60,61]. It has been reported that eHsp90α enhances the transcription and secretion of CXCL8 in prostate stromal fibroblasts to create an inflammatory stroma [62]. In the present study, we also found that the expression and secretion of CXCL8 were upregulated by eHsp90α to enhance lymphangiogenic activities of hLECs. Overall, the eHsp90α-LRP1-AKT-CXCL8 pathway is established in the lymphangiogenic process. Thorough interpretation of this molecular mechanism will further explain the biological significance of plasma Hsp90α as a diagnostic biomarker, and initially verify the viability of eHsp90α as a therapeutic target for pathogenic lymphangiogenesis in breast cancer.

## 4. Materials and Methods

### 4.1. Participant Enrollment and Quantitative Detection of Plasma Hsp90α

The breast cancer clinical trial (registered at ClinicalTrial.gov: NCT02324101) was conducted in accordance with Declaration of Helsinki. In the test cohort, 111 participants including 42 healthy individuals, 25 patients with at-risk breast diseases and 44 breast cancer patients were enrolled from June 2013 to June 2014. In the validation cohort, 1061 participants consisting of 328 healthy people, 139 patients with at-risk breast diseases and 594 breast cancer patients were recruited from June 2013 to September 2014. Detailed enrollment information was listed in Supplementary Figure S1 and Supplementary Table S1. This study was approved by the institutional ethics review committee of all participating hospitals. They are Zhejiang Province People’s Hospital, Fudan University Shanghai Cancer Center, Tianjin Medical University Cancer Institute and Hospital. Guided by each hospital’s regulation, written informed consents were received from all participants.

Three groups in each cohort were separated by clinicians. Healthy controls were chosen as without breast disease or other systematic diseases. At-risk breast diseases referred to mammary gland hyperplasia, mammary fibroadenoma, galactocele, mastitis and intraductal papilloma of the breast, which were diagnosed at least based on imaging methods. Breast cancer was diagnosed on the basis of imaging methods, and further confirmed by histopathology. Patients who had other solid tumors were excluded. Breast cancer patients were classified according to the TNM staging system, of which stages I and II breast cancer were recognized as early-stage breast cancer, and stages Ⅲ and Ⅳ were regarded as late-stage.

Peripheral blood samples were collected using EDTA-K2 anticoagulant tubes, and centrifuged at 3000 rpm for 10 min, then the upper plasma was stored at −80 °C for further use. Blood samples of hemolysis were excluded from this clinical trial. The plasma levels of Hsp90α were quantitatively measured using the commercial ELISA kit (Protgen, Yantai, China) with reference to the manufacturer’s instruction brochure.

### 4.2. Orthotopic Breast Cancer Mouse Models

All animal studies complied with the ARRIVE guidelines and were approved by the Institutional Animal Care and Use Committee of Tsinghua University, which received Animal Welfare Assurance from Office of Laboratory Animal Welfare (OLAW identification number: F16-00228; A5061-01). Animal studies were conducted as previously described [63]. To be short, MDA-MB-231/GFP cells (5 × 10^6^ cells per mouse) in the PBS and Matrigel mixed solution (1:1) were injected into the left fourth mammary fat pads of BLAB/c nude mice (female, 5 weeks). The mice were randomly divided into three groups (6 mice per group), and were respectively intravenously injected with PBS, mouse nonimmune IgG or Hsp90α neutralizing antibody one week after tumor implantation every two days. The injection dose of antibody is 5 mg per kg of mouse body weight. Similarly, MCF-7/GFP cells (1 × 10^6^ cells per mouse) were implanted into nude mice and grouped into three. The mice in each group were intravenously injected with PBS, BSA or recombinant Hsp90α protein one week after tumor implantation every two days. The injection dose of protein is 0.5 mg per kg of mouse body weight. Tumor volume was calculated based on the formula 1/2 × (length) × (width)^2^, and tumor growth curve was monitored at each time of intravenous treatment. Thirty days after implantation, all mice were sacrificed. The primary tumors and lymph nodes were dissected, weighed, photographed and examined by immunofluorescent staining. Lymphatic vessel densities (LYVE-1 positive areas) in primary tumors and metastasized cells (GFP positive signals) in lymph nodes were evaluated in 6 independent fields in different sections.

### 4.3. Cell Culture

Human dermal lymphatic endothelial cells (hLECs) were purchased from ScienCell Research Laboratories and cultured under the manufacturer’s instructions. Primary mouse lymphatic endothelial cells (mLECs) were isolated and cultivated as previously reported [64]. MDA-MB-231 and MCF-7 breast cancer cell lines were acquired from the American Type Culture Collection (Manassas, VA, USA) and cultured in conformity with their guidelines. Cells were preserved in 37 °C humidified incubators containing 5% CO_2_.

### 4.4. Antibodies and Reagents

Mouse monoclonal antibody against human Hsp90α was developed by our laboratory whose specificity and efficiency were verified [11,25]. Recombinant human Hsp90α protein was provided by Protgen (Yantai, China) [58,65]. Antibodies against LYVE-1 (ab14917), LRP1 (ab28320), CXCL8 (ab110727) and β-actin (ab8227) were from Abcam (Cambridge, UK). Recombinant human VEGFC protein (ab155739) was obtained from Abcam (Cambridge, UK). Human LRP1 neutralizing antibody (61065) was purchased from Progen (Heidelberg, Germany). CXCL8 (MAB208) neutralizing antibody was from R&D Systems (Minneapolis, MN, USA). Mouse IgG isotype control (5415S), rabbit IgG isotype control (2729S), and antibodies against AKT (9272), p-AKT (4060), ERK (4695), p-ERK (4377), LRP1 (64099S), 6 × His (12698S), and horseradish peroxidase (HRP)-conjugated anti-rabbit IgG (7074S) and HRP-conjugated anti-mouse IgG (7076S) antibodies were from Cell Signaling Technology (Danvers, MA, USA). TRITC-linked anti-rabbit (ZF-0317), FITC-linked anti-rabbit (ZF-0311) and TRITC-linked anti-mouse (ZF-0313) antibodies were from ZSGB-BIO Company (Beijing, China). Matrigel (354230) was purchased from BD Biosciences (San Jose, CA, USA). AKT inhibitor MK-2206 (S1078) and VEGFR-3 inhibitor SAR131675 (S2842) were from Selleck (Houston, TX, USA). DTBP (20665) was from Thermo Fisher Scientific (Waltham, MA, USA). Protein A-sepharose beads (11719408001) were from Roche (Basel, Switzerland).

### 4.5. Cell Viability Assay

LECs were seeded into 96-well plates (2 × 10^4^ cells per well) and cultivated overnight under normal conditions. Culture medium was then replaced with fresh medium containing 1% serum and recombinant Hsp90α protein at different dosages for 24 h. We also used the new medium with 10% serum to continue culturing LECs as a positive control. Cell viability was evaluated using CCK-8 (Dojindo, Tokyo, Japan) based on the manufacturer’s instructions. Each treatment was conducted in six wells and independent experiments were repeated in triplicate.

### 4.6. Cell Migration Assay

Cell migration ability in vitro was assessed with Millicell inserts (8 μm pore size; Merck Millipore, Darmstadt, Germany). First, 2 × 10^4^ mLECs or 6 × 10^4^ hLECs were seeded in 4% FBS-containing medium on the upper chambers, and indicated treatments in the same medium were added to the lower chambers. After incubation for 12 h, all inserts were fixed in 4% paraformaldehyde and stained with crystal violet. Migrated cells in each insert were counted in six random fields under an Olympus IX71 optical microscope (Tokyo, Japan), and three inserts were analyzed for each condition. Independent experiments were conducted three times.

### 4.7. Tube Formation Assay

The tube formation assay was performed in accordance with the previous study [66]. To be short, Matrigel was pre-added to form a solid layer at the bottom of a 96-well plate. The 2 × 10^4^ LECs were planted on the Matrigel layer with indicated reagents. After incubation for 6 h, tubular structures were photographed under an Olympus IX71 optical microscope. Six independent fields per well were captured and three wells were analyzed for each condition. Three independent experiments were repeated. Quantification was conducted with the Image Pro Plus 6.0 software (Media Cybernetics, Rockville, MD, USA).

### 4.8. Matrigel Plug Assay

Lymphatic vessel formation ability in vivo was evaluated using the Matrigel plug assay [23]. In brief, 0.4 mL of Matrigel solution containing needed regents were subcutaneously injected into the abdominal midline of BALB/c mice (female, 4–6 weeks old, 6 mice per group). After 8 days, mice were sacrificed. and the plugs were dissected for immunofluorescent staining. Lymphatic vessel formation ability was assessed with the LYVE-1 positive signals.

### 4.9. RNA Interference

siRNAs targeting mouse *Lrp1*, siRNA negative control and corresponding lentiviral shRNA were synthesized from GenePharma (Suzhou, China). siRNA and lentiviral shRNA sequences of *Lrp1* were listed in Supplementary Table S2. Guided by the manufacturer’s instruction, mLECs were transfected with siRNAs using lipofectamine 3000 (L3000001, Invitrogen, Carlsbad, CA, USA) in vitro. The efficacy of knockdown was estimated by immunoblotting 24–48 h after transfection. Lentivirus-delivered shRNA targeting *Lrp1* and shRNA negative control were used in the in vivo Matrigel plug assay (2.5 × 10^6^ U per plug).

### 4.10. Immunofluorescent (IF) Staining

For IF staining, frozen slices of Matrigel plugs, cultured cells, xenograft tumors and lymph nodes were fixed in cold acetone. The sliced samples were then blocked using 10% goat serum and incubated with appropriate primary antibodies overnight at 4 °C followed by corresponding fluorescent-conjugated secondary antibodies. DAPI was used to stain the nuclei. Six independent visual fields of each slice were photographed and analyzed with the Nikon A1 Confocal Microscope using Nikon image software (NIS-Elements AR 3.0, Tokyo, Japan).

### 4.11. Co-Immunoprecipitation Assay

To prove the interaction between eHsp90α and LRP1, hLECs were washed with PBS and treated with recombinant human Hsp90α protein (with 6 × His Tag, 200 ng/mL) for 15 min. After washing with PBS, protein–protein interactions were stabilized with the usage of 3 mg/mL dimethyl 3,3-dithiobispropionimidate (DTBP) for 2 h at 4 °C. hLECs were then collected and centrifuged at 3000 rpm for 5 min, and lysed with cell lysis buffer (20 mM Tris, 150 mM NaCl, 1% Triton X-100) supplemented with protease and phosphatase inhibitors. The supernatants were collected by centrifuging at 14,000× *g* for 5 min and incubated with indicated antibodies overnight at 4 °C with constant rotation. Protein A-sepharose beads were added into the complex, followed by rotation for another 4 h at 4 °C. The beads were then washed with lysis buffer and eluted in sample buffer (1% SDS, 1 mM DTT) by denaturing at 100 °C for 10 min. Western blotting analysis was further performed to detect target proteins. To explore the interaction between LRP1 and AKT, hLECs were washed with PBS and treated with or without recombinant human Hsp90α protein for 15 min. After washing with PBS, hLECs were collected and conducted co-immunoprecipitation assay with anti-LRP1 antibody.

### 4.12. Western Blotting

Protein levels were analyzed by Western blotting. Cells were harvested by scraping off culture plates and denatured with lysis buffer. Denatured samples were separated by SDS-PAGE gels and subsequently transferred to PVDF membranes (Merck Millipore, Darmstadt, Germany). After being blocked with 5% fat-free milk, the interest proteins in the membranes were probed with appropriate primary antibodies at 4 °C overnight, and then incubated with the corresponding HRP-labeled secondary antibodies for 1 h at room temperature. Finally, the target proteins were visualized using the chemiluminescence system (Thermo Fisher Scientific, Waltham, MA, USA) following the manufacturer’s procedure.

### 4.13. RNA Preparation and Data Analysis for RNA-Seq

RNA samples were extracted from hLECs with two treatments, one with MDA-MB-231 conditioned medium adding Hsp90α neutralizing antibody (named Hsp90α Ab group for short) and the other with the same medium adding antibody isotype (named IgG group for short). In each group, there were three parallels. The TRIzol reagent (Invitrogen, Carlsbad, CA, USA) was used to abstract RNA. The following RNA library construction and sequencing were performed on a BGISEQ-500 platform in Beijing Genomic Institution (BGI, Shenzhen, China).

To analyze gene expression, the matched reads were calculated and normalized to TPM (short for transcripts per million) [67]. Based on the prerequisite of *p*-value < 0.05, we chose genes satisfying the condition that value of Fold Change (Hsp90α Ab group *versus* IgG group) > 1.2 (upregulated by Hsp90α Ab) or Fold Change < 1/1.2 (downregulated by Hsp90α Ab) as differentially expressed genes (DEGs). A volcano plot was drawn to display DEGs using Origin software. Gene Ontology (GO) analysis of DEGs was conducted on Metascape (https://metascape.org/). Genes involved in the positive or negative regulation of angiogenesis and lymphangiogenesis were acquired from AmiGo 2 (http://amigo.geneontology.org/) and separately intersected with the downregulated or upregulated DEGs by Hsp90α Ab using a Venn diagram.

### 4.14. Reverse Transcription and Quantitative Real-Time PCR (qRT-PCR)

cDNA was synthesized using the First Strand cDNA Synthesis Kit (Thermo Fisher Scientific, Waltham, MA, USA) from total RNA. qRT-PCR was further conducted to quantify levels of gene expression with TransStartGreen qPCR SuperMix (TransGen Biotech, Beijing, China) in a Mx3000P system (Stratagene, La Jolla, CA, USA). Relative quantification of mRNA levels was normalized by β-actin and achieved with the 2^−ΔΔCt^ method. Independent experiments were performed in triplicate. The sequence information for all primers is listed in Supplementary Table S3.

### 4.15. Quantification of CXCL8 by ELISA

Human LECs were cultured in complete medium with 10% FBS until the confluence reached 80%. The culture medium was then replaced with fresh medium containing 1% FBS, in which recombinant Hsp90α or control BSA was administrated. After 16 h, the supernatants were harvested from each culture and stored at −80 °C for further detection. The concentrations of CXCL8 were quantified using the human CXCL8/IL-8 ELISA Kit (D8000C, R&D Systems, Minneapolis, MN, USA).

### 4.16. Statistical Analysis

Clinical data were presented as mean ± SEM. Comparisons were performed in GraphPad Prism software using unpaired, non-parametric Mann–Whitney test. Experimental data were summarized as mean ± SD. Statics between groups were compared in GraphPad Prism software using unpaired Student’s *t* tests. *p*-value < 0.05 was considered statistically significant. Receiver operating characteristics (ROC) curves were used to assess the sensitivity, specificity, and respective areas under the curve (AUC) with 95% confidence interval (CI). The optimum cutoff of plasma Hsp90α value for breast cancer auxiliary diagnosis was based on the Youden index (J) method [68].

## 5. Conclusions

Our clinical trial qualifies plasma Hsp90α as a novel diagnostic biomarker for breast cancer and early-stage breast cancer. Moreover, levels of plasma Hsp90α in breast cancer patients are positively associated with regional lymph nodes metastasis. In the experimental study, we reveal for the first time the role of eHsp90α as a pro-lymphangiogenic molecule, which induces primary tumor lymphangiogenesis in vivo and promotes lymphangiogenic activities of LECs in vitro. Mechanistically, eHsp90α signals to the AKT pathway through LRP1 to promote the secretion of CXCL8 in the lymphangiogenic process. Our work on eHsp90α provides not only an auxiliary diagnostic biomarker for breast cancer, but also a potential target for therapeutic intervention in pathogenic lymphangiogenesis.

## Figures and Tables

**Table 1:** Diagnostic performance of plasma Hsp90α in the detection of breast cancer.

	AUC (95% CI)	Cutoff (ng/mL)	Sensitivity (%)	Specificity (%)
**Test Cohort**				
BC vs. HC + AR	0.8351 (0.7587–0.9115)	49.11	79.55	77.61
BC vs. AR	0.7055 (0.5761–0.8349)	55.26	72.73	72.00
BC vs. HC	0.9123 (0.8530–0.9716)	43.89	84.09	83.33
**Validation Cohort**				
BC vs. HC + AR	0.7973 (0.7704–0.8242)	50.29	74.41	71.95
BC vs. AR	0.6249 (0.5749–0.6750)	59.63	60.94	60.43
BC vs. HC	0.8703 (0.8468–0.8939)	46.09	80.13	81.40
Early-BC vs. HC + AR	0.7828 (0.7542–0.8114)	50.29	72.33	71.95

BC indicates breast carcinoma. HC is short for healthy control. AR represents at-risk (non-cancerous) breast diseases, including mammary gland hyperplasia, mammary fibroadenoma, galactocele, mastitis and intraductal papilloma of the breast. Early-BC indicates patients with early-stage (stages I and II) breast cancer. AUC represents area under curve. CI is short for confidence interval.

## Data Availability

All data generated or analyzed during this study are included in this published article and its Supplementary Materials.

## Supplementary Materials

The following are available online at https://www.mdpi.com/article/10.3390/ijms22147747/s1.

## Abbreviations

Hsp90α	Heat shock protein 90alpha
eHsp90α	Extracellular Hsp90α
LYVE-1	Lymphatic vessel hyaluronan receptor-1
LECs	Lymphatic endothelial cells
hLECs	Human LECs
mLECs	Mouse LECs
CM	Conditioned medium
LRP1	Low-density lipoprotein receptor-related protein 1
VEGF-C/D	Vascular endothelial growth factor C/D
VEGFR-3	Vascular endothelial growth factor receptor 3
AKT	Protein kinase B
ERK	Extracellular regulated protein kinases
CXCL8	C-X-C motif chemokine ligand 8
