# Supplementary material for: Extracellular Hsp90α Promotes Tumor Lymphangiogenesis and Lymph Node Metastasis in Breast Cancer
Source: Int J Mol Sci. 2021 Jul 20;22(14):7747. doi: 10.3390/ijms22147747 (PMC8305043; doi:10.3390/ijms22147747)
Supplement: Supplementary file 1 [file ijms-22-07747-s001.zip › Supplementary Figures-20210705.pdf]

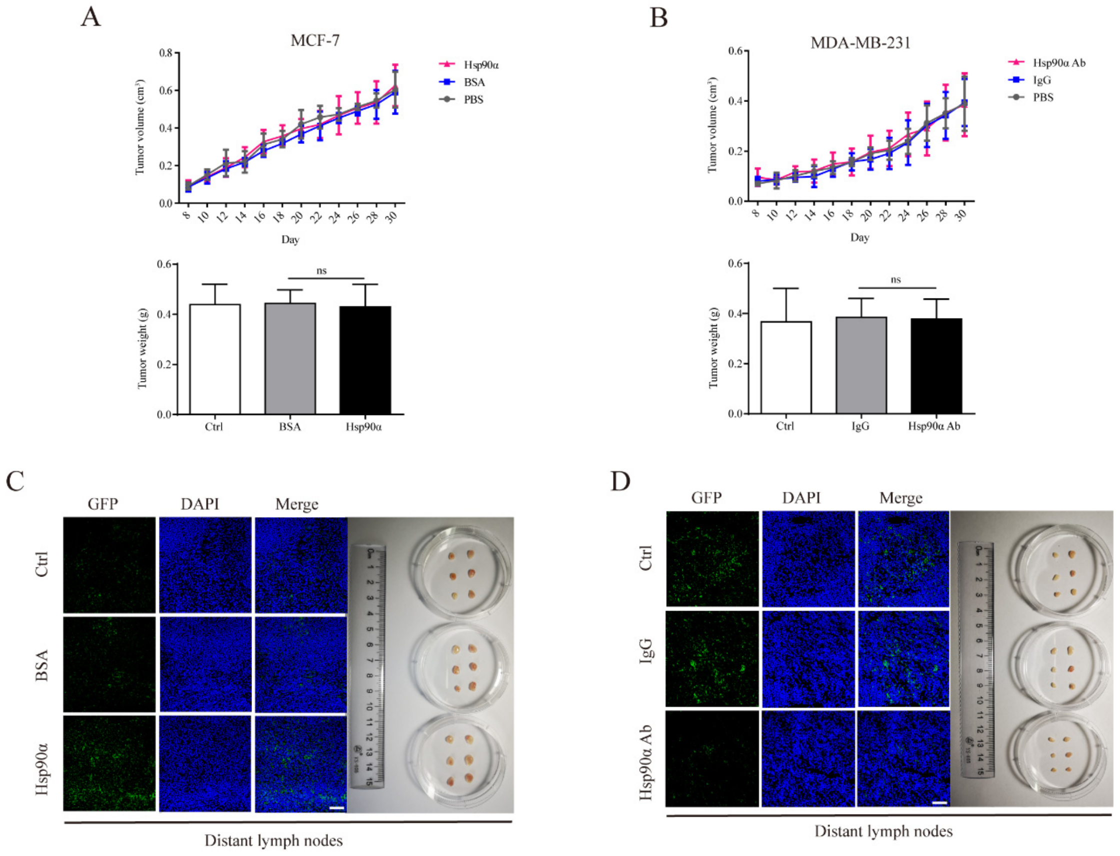

Figure S1. The influence of eHsp90*α* on breast cancer growth and distant lymph node metastasis *in vivo*. (A, B) Tumor volumes of MCF-7/GFP tumor-bearing mice in different groups (A, top) and MDA-MB-231/GFP tumor-bearing mice in different groups (B, top) were monitored at each time of intravenous treatment. Tumor weights of MCF-7/GFP mouse models (A, bottom) and MDA-MB-231/GFP mouse models (B, bottom) were measured at the thirty days when mice were sacrificed. (C) Representative IF images of metastatic tumor cells (GFP labeled) in distant lymph nodes removed from MCF-7/GFP tumor-bearing mice in different groups (left) and photographs of dissected distant lymph nodes (right). Scale bar, 50 μm. (D) Representative IF images of metastatic tumor cells (GFP labeled) in distant lymph nodes removed from MDA-MB-231/GFP tumor-bearing mice in different groups (left) and photographs of dissected distant lymph nodes (right). Scale bar, 50 μm. Data are shown as mean ± SD. n = 6.

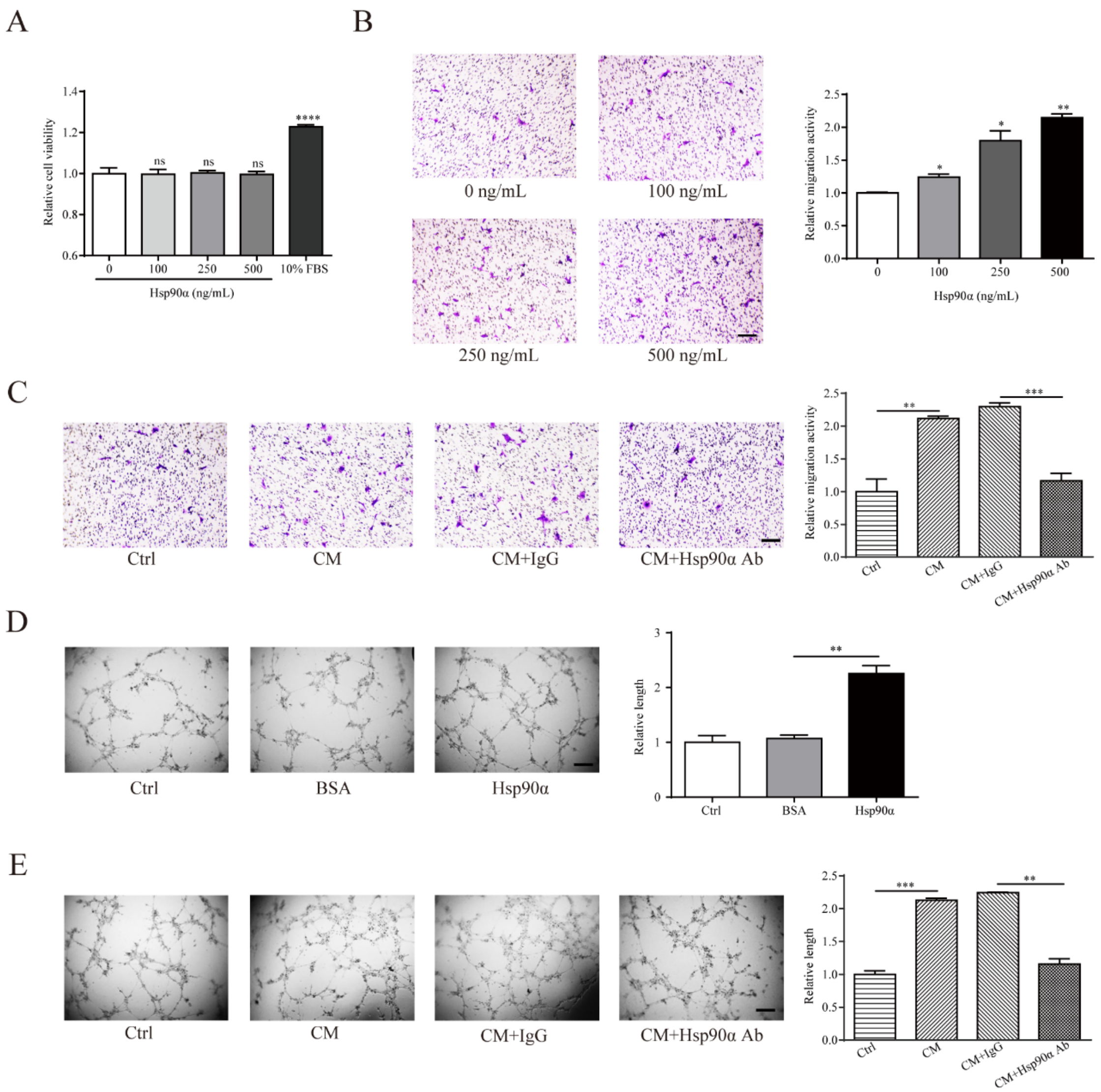

Figure S2. The effect of eHsp90α on the migration and tube formation abilities of mLECs. (A) Quantification results of the cell viability of mLECs treated with different dosages of recombinant Hsp90α protein in the culture medium containing 1% FBS. Medium with 10% FBS was used as the positive control. (B, C) Representative images and quantification results of the cell migration ability of mLECs treated with recombinant Hsp90α protein (B) or with MDA-MB-231 CM, which was pre-mixed with Hsp90α neutralizing antibody or control IgG (C). Scale bar = 200 μm. (D, E) Representative images and quantification results of the tube formation ability of mLECs treated with recombinant Hsp90α protein (D) or with MDA-MB-231 CM, which was pre-incubated with Hsp90α neutralizing antibody or control IgG (E). Concentration of recombinant Hsp90α protein or Hsp90α neutralizing antibody in the migration and tube formation assay is 500 ng/mL. Scale bar = 500 μm. Data from three independent experiments are displayed as the mean ± SD. *$P < 0.05$; **$P < 0.01$; ***$P < 0.001$; ****$P < 0.0001$.

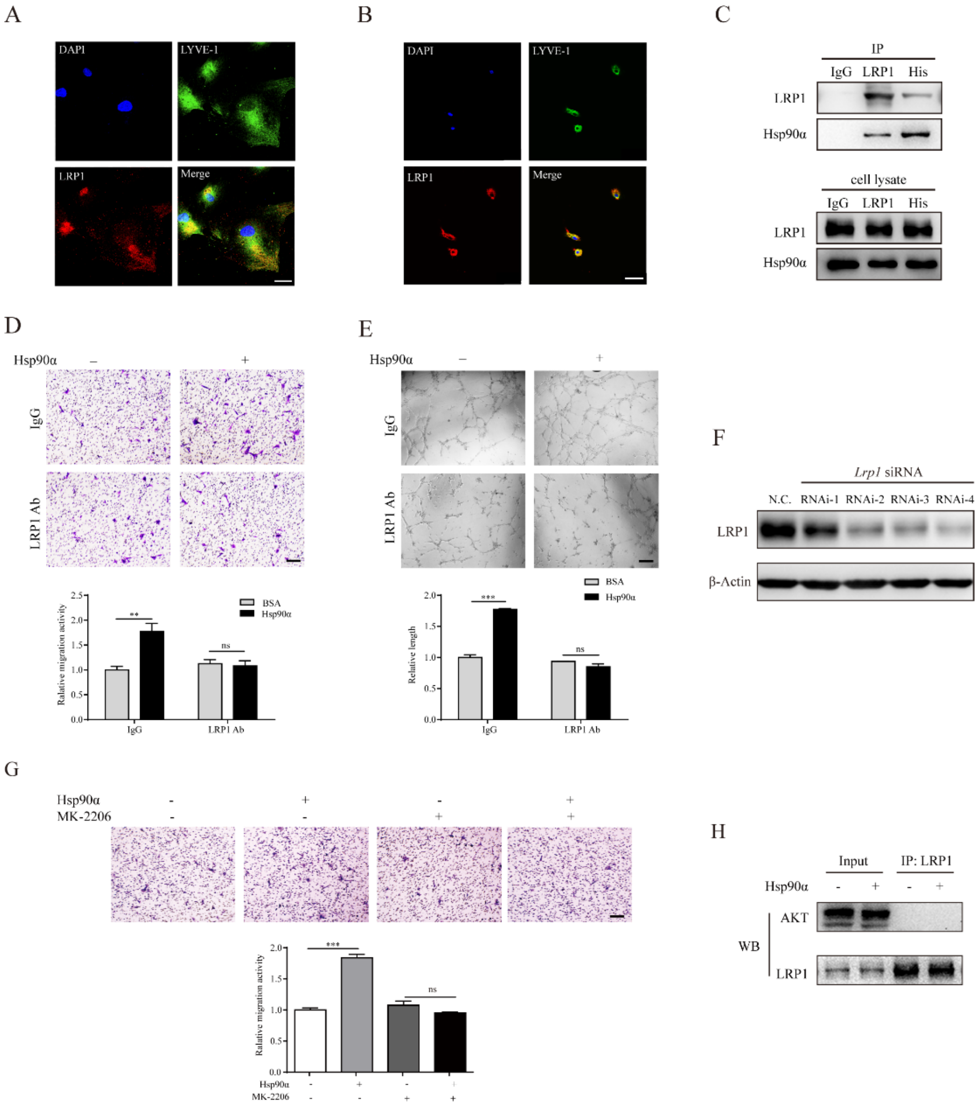

Figure S3. The role of LRP1-AKT in the eHsp90α-induced lymphangiogenic activities. (A, B) Confirmation of LRP1 in the cultured hLECs (A) and mLECs (B) *in vitro* by immunofluorescence. Scale bar, 20 μm for hLECs and 50 μm for mLECs. (C) hLECs were incubated with recombinant human Hsp90α protein (with 6×His Tag) for 15 min, and protein-protein interaction was stabilized using 3 mg/ml DTBP. Cell lysate was immunoprecipitated with anti-IgG, anti-6×His or anti-LRP1 antibodies, and underwent western blotting with anti-LRP1 or anti-Hsp90α antibodies. Total cell lysate was used as input. (D, E) Representative images and quantification results of the migration ability (D) and tube formation ability (E) of cells induced by recombinant Hsp90α protein (500 ng/mL) when mLECs were pretreated with LRP1 neutralizing antibody (200 ng/mL). Scale bar, 200 μm for migration assay and 500 μm for the tube formation assay. (F) The knockdown efficacy of LRP1 in mLECs using four *Lrp1* siRNAs was determined by Western blotting. (G) Representative images and quantification results of the cell migration ability of MK-2206-pretreated mLECs induced by recombinant Hsp90α protein. Scale bar, 200 μm. (H) hLECs were incubated with or without recombinant human Hsp90α protein for 15 min. The cell lysate was immunoprecipitated with anti-LRP1 antibody, and underwent western blotting with anti-LRP1 and anti-AKT antibodies. Total cell lysate was used as input. Data are represented as mean ± SD. $^{**}P < 0.01$; $^{***}P < 0.001$.

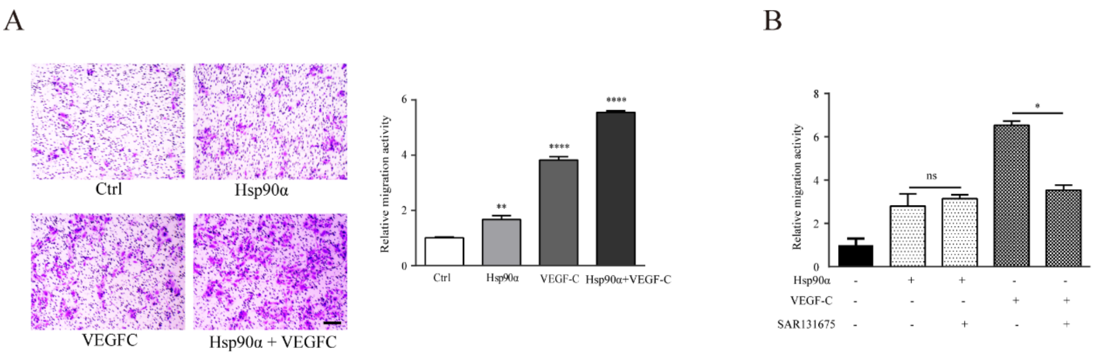

Figure S4. The relationship of eHsp90*α*/LRP1 and VEGF-D/VEGFR3 in the lymphangiogenic migration of hLECs. (A) Representative images and quantification results of the migration activities of hLECs treated with recombinant Hsp90*α* protein (200 ng/mL) or VEGF-C (100 ng/mL) or their combination. (B) Quantification results of the migration activities of hLECs induced by Hsp90*α* or VEGF-C in the presence or absence of VEGFR-3 inhibitor SAR131675 (20 nM). Data are represented as mean ± SD. *$P < 0.05$; **$P < 0.01$; ***$P < 0.001$; ****$P < 0.0001$.

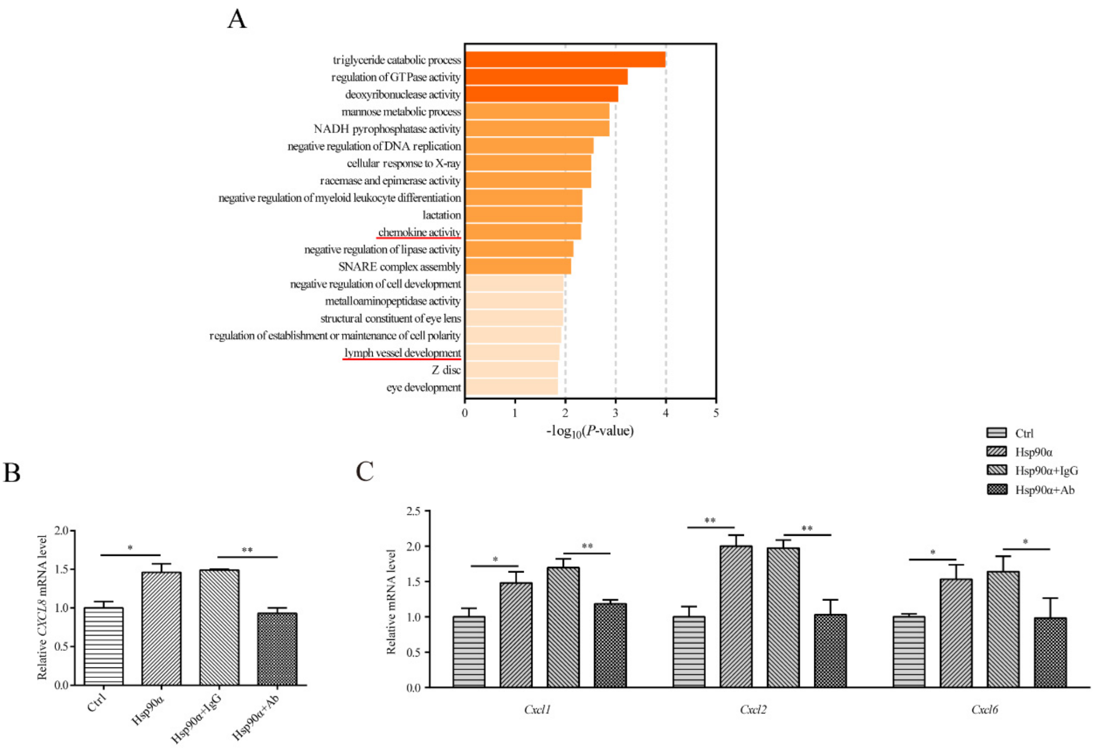

Figure S5. Gene Ontology and qRT-PCR analysis of differentially expressed genes. (A) DEGs were conducted the Gene Ontology (GO) analysis using Metascape. The top twenty GO enriched terms were sorted by descending order of *P*-value. (B, C) mRNA levels of *CXCL8* in hLECs (B) and *Cxcl1/2/6* in mLECs (C) stimulated by recombinant Hsp90*α* protein in the presence of Hsp90*α* neutralizing antibody or control IgG. Data are shown as mean ± SD. **P* < 0.05; ***P* < 0.01.

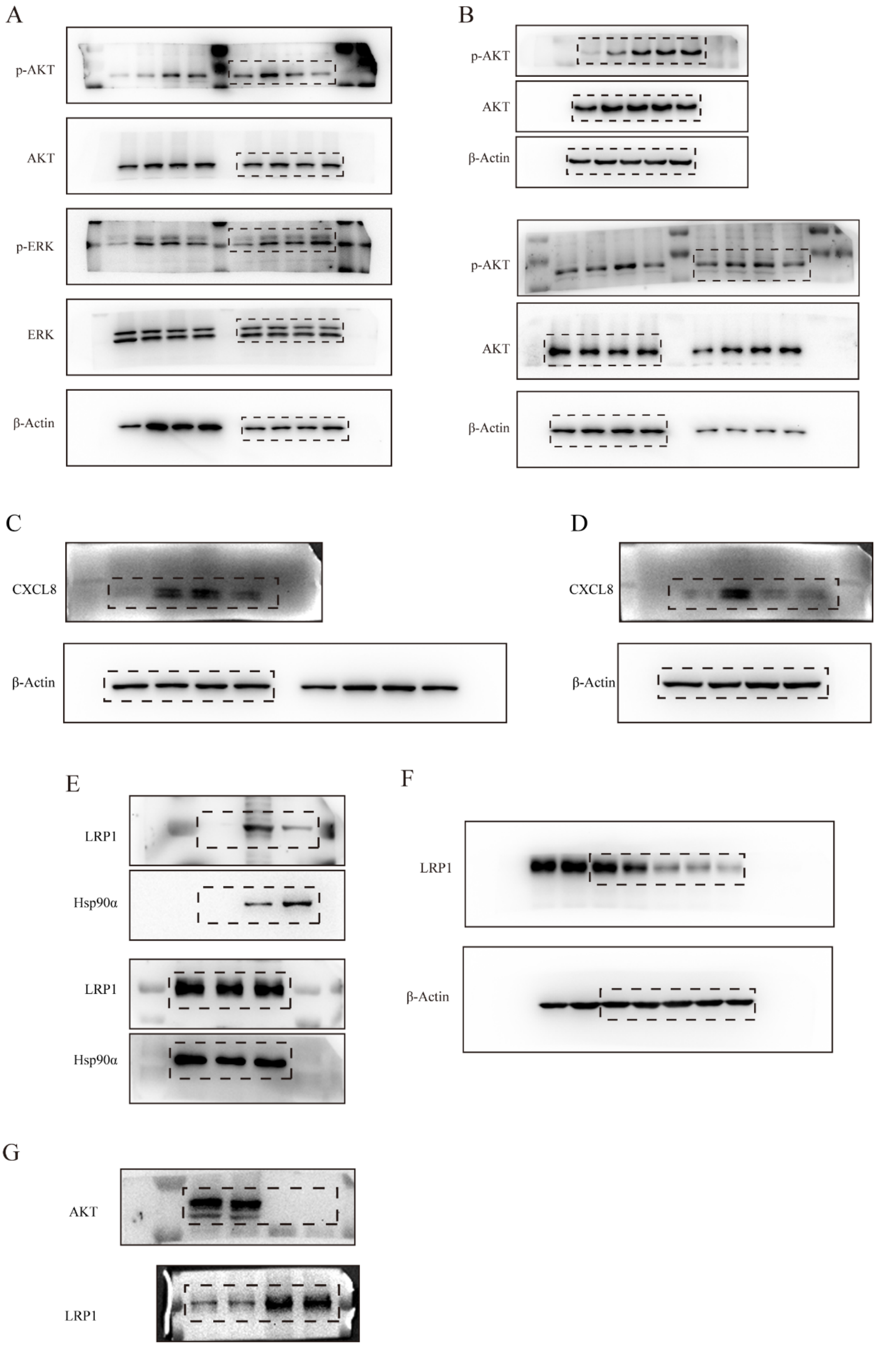

Figure S6. Uncropped Western blotting results in Figures. (A, B) Uncropped Western blotting results in Figure 4E (A) and Figure 4F (B). (C, D) Uncropped Western blotting results in Figure 5E (C) and Figure 5F (D). (E, F, G) Uncropped Western blotting results in Supplementary Figure S3C (E), S3F (F) and S3H (G). Membranes were often cut to enable blotting with multiple primary antibodies. Samples in the dotted box is the target samples.
