# Supplementary material for: Extracellular Hsp90α Promotes Tumor Lymphangiogenesis and Lymph Node Metastasis in Breast Cancer
Source: Int J Mol Sci. 2021 Jul 20;22(14):7747. doi: 10.3390/ijms22147747 (PMC8305043; doi:10.3390/ijms22147747)
Supplement: Supplementary file 1 [file ijms-22-07747-s001.zip › Supplementary Tables-20210705.pdf]

Table S1. Participant distribution in the clinical trial

Group	Hospital	No. of participants
**Test cohort**		
Healthy individuals	Zhejiang Province People's Hospital	42
Patients with at-risk breast diseases	Fudan University Shanghai Cancer Center	25
Breast cancer patients	Tianjin Medical University Cancer Institute and Hospital	44
**Validation cohort**		
Healthy individuals	Zhejiang Province People's Hospital	328
Patients with at-risk breast diseases	Fudan University Shanghai Cancer Center	139
Breast cancer patients	Fudan University Shanghai Cancer Center	594

Table S2. siRNA and lentiviral shRNA sequences of *Lrp1*

Name	siRNA sequence (5'-3')	Lentiviral shRNA sequence (5'-3')
Lrp1-RNAi-1	GCGUGGUGUUCUGGUAUAATT	
Lrp1-RNAi-2	GCCCAUUGGAUGAGUUUCATT	
Lrp1-RNAi-3	CCACCUGCUAUGAGUUUAATT	CCACCTGCTATGAGTTTAA
Lrp1-RNAi-4	GUCGCUGUGUAUCCAACAUTT	GTCGCTGTGTATCCAACAT

Table S3. Primer sequences for qRT-PCR

Gene symbol	Forward primer (5'-3')	Reverse primer (5'-3')
ACTB	CATGTACGTTGCTATCCAGGC	CTCCTTAATGTCACGCACGAT
CCL24	GGAGTGGGTCCAGAGGTACAT	CAGGTGGTTTGGTTGCCAG
CXCL8	ACTGAGAGTGATTGAGAGTGGAC	AACCCTCTGCACCCAGTTTTC
HDAC9	AGTAGAGAGGCATCGCAGAGA	GGAGTGTCTTTCGTTGCTGAT
Cxcl1	CTGGGATTCACCTCAAGAACATC	CAGGGTCAAGGCAAGCCTC
Cxcl2	CCAACCACCAGGCTACAGG	GCGTCACACTCAAGCTCTG
Cxcl6	TCCAGCTCGCCATTCATGC	TTGCGGCTATGACTGAGGAAG

Table S4. Concentration of plasma Hsp90α in test cohort

Group	No.	Median (IQR) (ng/mL)	Mean (SD) (ng/mL)
HC	42	33.60 (25.43-39.65)	33.98 (13.44)
AR	25	47.77 (39.17-62.08)	57.85 (29.94)
HC+AR	67	38.09 (28.02-47.96)	42.89 (23.95)
BC	44	68.83 (51.15-96.93)	84.41 (54.77)

IQR, interquartile range; SD, standard deviation; HC, healthy control; AR, at-risk breast diseases (non-cancerous); HC+AR, non-cancer control; BC, breast cancer.

Table S5. Concentration of plasma Hsp90α in validation cohort

Group	No.	Median (IQR) (ng/mL)	Mean (SD) (ng/mL)
HC	328	32.24 (22.53-43.14)	35.06 (18.73)
AR	139	55.17 (41.31-69.39)	62.81 (34.09)
HC+AR	467	38.44 (24.78-53.95)	43.32 (27.41)
BC	594	66.50 (49.81-93.25)	81.87 (77.01)
Early-BC	524	64.53 (48.51-87.28)	72.11 (36.50)
Late-BC	70	100.70 (64.98-152.10)	154.90 (186.40)

IQR, interquartile range; SD, standard deviation; HC, healthy control; AR, non-cancerous breast diseases; HC+AR, non-cancer control; BC, breast cancer; Early-BC, early-stage (stage I and II) breast cancer, Late-BC, late-stage (stage III and IV) breast cancer.

Table S6. Plasma Hsp90α concentration of breast cancer patients in different clinical stages of regional lymph node metastasis

Group	No.	Median (IQR) (ng/mL)	Mean (SD) (ng/mL)
HC+AR	467	38.44 (24.78-53.95)	43.32 (27.41)
cN0	393	64.13 (47.68-85.92)	70.74 (35.29)
cN1	147	69.70 (51.73-93.88)	80.94 (46.98)
cN2	13	88.00 (72.42-118.90)	98.05 (47.30)
cN3	12	88.29 (59.55-147.40)	115.44 (81.88)
cN1+cN2+cN3	172	71.63 (52.11-97.65)	84.64 (50.64)

IQR, interquartile range; SD, standard deviation; HC+AR, non-cancer control. cN0 stage is defined as no regional lymph node metastases (by imaging or clinical examination). cN1 stage is defined as metastases to movable ipsilateral level I and II axillary lymph node(s). cN2 stage is defined as metastases in ipsilateral level I and II axillary lymph nodes that are clinically fixed or matted; or in ipsilateral internal mammary lymph nodes in the absence of axillary lymph node metastases. cN3 stage is defined as metastases in ipsilateral infraclavicular (level III axillary) lymph node(s) with or without level I and II axillary lymph node involvement; or in ipsilateral internal mammary lymph node(s) with level I and II axillary lymph node metastases; or metastases in ipsilateral supraclavicular lymph node(s) with or without axillary or internal mammary lymph node involvement. The degree of regional lymph node metastasis follows: cN0 < cN1 < cN2 < cN3. cN1+cN2+cN3 group represents patients with regional lymph node metastasis, including cN1, cN2 and cN3 categories.

Table S7. Detailed information of the top 50 down-regulated or up-regulated DEGs

Gene symbol	log_2(fold change)	*P*-value	Gene symbol	log_2(fold change)	*P*-value
PHOSPHO2-KLHL23	-2.82	0.04	LOC101928589	1.98	0.05
LOC100996911	-1.23	0.03	PCDHB5	0.95	0.02
TMEM45A	-1.00	0.03	FAM72A	0.75	0.02
HIST1H3E	-0.97	0.02	CA12	0.70	0.01
BCL2L10	-0.94	0.02	FUOM	0.67	0.00
TMEM189-UBE2V1	-0.87	0.04	GP1BB	0.65	0.04
DNASE1L3	-0.78	0.03	TBC1D3	0.65	0.02
HERC5	-0.77	0.03	EFCAB8	0.63	0.03
CBX8	-0.76	0.02	NRROS	0.60	0.00
HSF4	-0.76	0.01	RGS20	0.60	0.04
GOLGA8T	-0.72	0.02	CCDC157	0.59	0.03
CLEC12B	-0.70	0.01	ZNF662	0.55	0.01
PNPLA3	-0.69	0.02	PTPRCAP	0.52	0.00
DRAXIN	-0.67	0.01	LOC107986805	0.51	0.00
TNIP3	-0.66	0.04	RELL2	0.50	0.01
POPDC3	-0.66	0.03	HYKK	0.49	0.03
LOC105373132	-0.63	0.04	SYCE1L	0.48	0.03
ZNF485	-0.62	0.03	ARHGEF19	0.48	0.03
ZNF382	-0.62	0.00	MLANA	0.46	0.03
IFIT2	-0.61	0.01	LLGL2	0.44	0.04
EPSTI1	-0.59	0.04	LOC100505549	0.43	0.04
ZNF441	-0.58	0.05	MFSD13A	0.42	0.03
ERCC6L2	-0.58	0.00	NUDT17	0.41	0.02
ZFP2	-0.56	0.02	USP44	0.41	0.03
ANKHD1	-0.55	0.02	PRELID3A	0.41	0.04
SERHL2	-0.53	0.02	UCP3	0.40	0.02
PCDHB2	-0.52	0.04	HOOK2	0.40	0.01
CCL7	-0.51	0.02	ZNF575	0.39	0.05
ZNF121	-0.51	0.04	RILP	0.39	0.03
TIGD6	-0.48	0.04	SAA2	0.38	0.04
SLC41A2	-0.47	0.04	ZSWIM9	0.38	0.04
WDR83	-0.47	0.01	GALM	0.38	0.01
KIAA1328	-0.47	0.04	STX1A	0.38	0.01
VAMP4	-0.45	0.01	NR2F1	0.38	0.02

Table S7 (continued)

**Gene symbol**	**log$_2$(fold change)**	***P*-value**	**Gene symbol**	**log$_2$(fold change)**	***P*-value**
C22orf23	-0.44	0.04	CHAC2	0.36	0.00
LOC100506248	-0.43	0.05	SOCS2	0.36	0.01
SYT15	-0.42	0.04	SGK494	0.35	0.03
BFSP1	-0.41	0.00	THG1L	0.35	0.03
**CXCL8**	**-0.41**	**0.04**	BNIPL	0.34	0.05
EIF4E3	-0.40	0.01	ECHDC2	0.33	0.03
GSAP	-0.40	0.05	DFFB	0.32	0.03
C12orf66	-0.40	0.05	PMM1	0.31	0.01
LNPEP	-0.40	0.02	LOC100652901	0.30	0.05
CRYBG3	-0.40	0.01	ITPA	0.29	0.05
ZNF846	-0.39	0.02	NEK5	0.29	0.02
**CCL24**	**-0.39**	**0.05**	LOC107984026	0.29	0.03
ABHD12B	-0.38	0.03	ZNHIT2	0.29	0.01
TPBG	-0.37	0.05	PILRB	0.29	0.03
PPP1R12B	-0.37	0.05	AVPI1	0.28	0.05
PLLP	-0.37	0.04	LIPE	0.28	0.00
